# Risk of Clade II Mpox Associated with Intimate and Nonintimate Close Contact Among Men Who Have Sex with Men and Transgender Adults — United States, August 2022–July 2023

**DOI:** 10.15585/mmwr.mm7340a2

**Published:** 2024-10-10

**Authors:** Anna N. Chard, Alexandra F. Dalton, Alpha Oumar Diallo, Danielle L. Moulia, Nicholas P. Deputy, Italo B. Zecca, Laura A.S. Quilter, Rachel E. Kachur, Andrea M. McCollum, Jemma V. Rowlands, Amber N. Britton, Rebecca Fisher, Shua J. Chai, Erin Licherdell, William L. Still, Adeline L. Morris, Jessica L. Castilho, Tiffanie M. Markus, Allison S. Morrow, Phoebe Danza, AmberJean P. Hansen, Sophia Ibrahim Ali, Christopher W. Wegner, Robyn Weber, Gabriela S. Betancourt, Jennifer Zipprich, Melissa Sutton, Preeti Pathela, Sam Hawkins, Karen A. Wendel, Leora R. Feldstein

**Affiliations:** ^1^Influenza Division, National Center for Immunization and Respiratory Diseases, CDC; ^2^Coronavirus and Other Respiratory Viruses Division, National Center for Immunization and Respiratory Diseases, CDC; ^3^Division of Viral Diseases, National Center for Immunization and Respiratory Diseases, CDC; ^4^Division of Birth Defects and Infant Disorders, National Center on Birth Defects and Developmental Disabilities; ^5^Division of High-Consequence Pathogens and Pathology, National Center for Emerging and Zoonotic Infectious Diseases, CDC; ^6^Epidemic Intelligence Service, CDC; ^7^Division of STD Prevention, National Center for HIV, Viral Hepatitis, STD, and TB Prevention, CDC; ^8^New York State Department of Health; ^9^Georgia Emerging Infections Program, Georgia Department of Health; ^10^Emory University School of Medicine, Atlanta, Georgia; ^11^Los Angeles County Department of Public Health, Los Angeles, California; ^12^California Emerging Infections Program, Oakland, California; ^13^Career Epidemiology Field Officer Program, CDC; ^14^University of Rochester School of Medicine and Dentistry, Rochester, New York; ^15^DC Health, Washington, DC; ^16^The Connecticut Emerging Infections Program, Yale School of Public Health, New Haven, Connecticut; ^17^Department of Health Policy, Vanderbilt University Medical Center, Nashville, Tennessee; ^18^Minnesota Department of Health; ^19^Colorado Department of Public Health and Environment; ^20^New York City Department of Health and Mental Hygiene; ^21^Public Health Division, Oregon Health Authority; ^22^Denver Health, Denver, Colorado; ^23^Innovate Division, Center for Forecasting and Outbreak Analytics, CDC.

**Summary**

**What is already known about this topic?**

Monkeypox virus can spread through intimate or close contact with a person with mpox.

**What is added by this report?**

Among men who have sex with men and transgender persons who reported close contact with a person with mpox, condomless receptive anal sex was associated with approximately five times the odds of mpox after controlling for mpox vaccination, sociodemographic characteristics, and concurrent close contact behaviors.

**What are the implications for public health practice?**

The findings in this report underscore the importance of ongoing multifaceted mpox prevention activities, including mpox vaccination and education on safer sex practices, to reduce the spread of mpox.

## Abstract

A global outbreak of clade II mpox associated with sexual contact, disproportionately affecting gay, bisexual, and other men who have sex with men (MSM), has been ongoing since May 2022. Information on types of contact most associated with transmission is limited. This report used data from a multijurisdictional vaccine effectiveness case-control study of sexually active persons aged 18–49 years who identified as MSM or transgender, collected during August 2022–July 2023. Odds of mpox associated with selected types of intimate and nonintimate close contact with a person with mpox were estimated. Among 457 case-patients and 1,030 control patients who met minimum data requirements, 150 (32.8%) case-patients and 57 (5.5%) control patients reported close contact with a person with mpox and were included in this analysis. Adjusted odds of mpox were 5.4 times as high among those who reported having condomless receptive anal sex with a person with mpox, compared with participants who reported close contact with a person with mpox and no condomless receptive anal sex with that person (OR = 5.4; p = 0.031). Although the mpox vaccine is highly effective, vaccination coverage remains low; a multifaceted approach to prevention remains important and should include vaccination promotion, safer sex practices, and increasing awareness that mpox continues to circulate.

## Introduction

In May 2022, an unprecedented worldwide outbreak of clade II mpox, caused by monkeypox virus (MPXV), was detected among persons in countries with no history of sustained community transmission. In the United States and worldwide, the ongoing outbreak has been associated with sexual contact, and has disproportionately affected gay, bisexual, and other men who have sex with men (MSM) (*1*,*2*). Transmission via other forms of nonintimate close contact, such as contaminated household objects and surfaces, is rare, but has also been reported (*3*). The risk of mpox associated with selected intimate and nonintimate close contact behaviors during the outbreak was estimated among MSM and transgender persons aged 18–49 years using data from 12 U.S. jurisdictions.*

## Methods

### Study Design and Data Collection

A secondary analysis was conducted using data previously collected for a vaccine effectiveness (VE) case-control study using patient self-reported survey data and jurisdiction-reported data from 12 U.S. jurisdictions (*4*). In the VE study, case-patients (those with a confirmed or probable MPXV or orthopoxvirus diagnosis on or after August 19, 2022) were identified through jurisdiction health departments’ case registries. Control patients (persons with a health care encounter at the clinic on or after August 19, 2022, and who did not report an mpox diagnosis) were identified through active and passive recruitment approaches in sexual health, HIV care, or HIV preexposure prophylaxis (PrEP) clinics in each jurisdiction. During recruitment, control patients were frequency matched to case-patients based on timing of index event (test result or medical encounter date) and geographic region.

Case- and control patients were eligible to participate if they were sexually active,^†^ aged 18–49 years, and identified as MSM^§^ or transgender. Eligible participants completed a survey online or by telephone in English or Spanish. The survey included questions about demographic characteristics, mpox vaccination history (verified using jurisdiction vaccination registries, where available), mpox diagnosis, and mpox exposure history^¶^ anchored to an index date, defined as the date of receipt of a positive test result (case-patients) or clinic visit (control patients). Participants who reported having close contact with a person with diagnosed mpox or a person with symptoms consistent with mpox but who did not receive a diagnosis (hereafter referred to as close contact with a person with mpox) were asked follow-up questions about specific types of intimate** and nonintimate^††^ contact. Survey responses were recorded in REDCap (version 13.1.26; Vanderbilt University).

### Statistical Methods

The analytic sample was restricted to case- and control patients from the VE study who reported close contact with a person with mpox. Multilevel logistic regression models were used to examine the unadjusted and adjusted odds of select types of intimate and nonintimate contact and case- or control patient status. The unadjusted models examined demographic characteristics (age, race, and gender identity), a composite variable of HIV status and HIV PrEP or treatment (i.e., antiretroviral [ARV]) use and adherence, presence of immunocompromising conditions or medications,^§§^ number of recent sexual partners,^¶¶^ recent sexually transmitted infection diagnosis, mpox vaccination status,*** and month of index event, and included a random intercept for jurisdiction to account for clustering. Adjusted models included demographic and health history covariates with p<0.05 in the unadjusted models, index month, and all reported close contact behaviors. Analyses were conducted using Stata (version 16; StataCorp). This activity was reviewed by CDC, deemed not research, and was conducted consistent with applicable federal law and CDC policy.^†††^

## Results

Among the 1,487 eligible survey respondents from the VE study (457 case-patients and 1,030 control patients), 207 (13.9%; 150 case-patients and 57 control patients) reported close contact with a person with mpox and were included in this analysis. Compared with control patients, case-patients were slightly older (aged 35.5 years versus 33 years) and had fewer recent sexual partners (two versus three) (Table 1). A lower proportion of case-patients (45.8%) than control patients (76.9%) reported using HIV PrEP, and a higher proportion of case-patients (72.9%) than control patients (23.5%) were not vaccinated against mpox.

**TABLE 1:** Selected characteristics of mpox case-patients and control patients reporting close contact with a person with mpox — 12 jurisdictions, United States,* August 2022–July 2023

Characteristic	Case-patient,^† ^no. (%)^§ ^n = 150	Control patient,^¶^ no. (%)^§ ^n = 57	p*-*value**
**Median age, yrs (IQR)**	35.5 (31–42)	33 (29–38)	0.036
**Race and ethnicity** ^††^			
Black or African American, non-Hispanic	46 (30.7)	15 (26.3)	0.168
White, non-Hispanic	45 (30.0)	25 (43.9)	
Hispanic or Latino	47 (31.3)	11 (19.3)	
Other, non-Hispanic	12 (8.0)	6 (10.5)	
**Gender identity**			
Male	140 (93.3)	52 (91.2)	0.135
Transgender female	7 (4.7)	1 (1.8)	
Transgender male	—	—	
Another gender identity	3 (2.0)	4 (7.0)	
**Median (IQR) number of sexual partners^§§^**	2 (1–4)	3 (2–5)	0.013
**HIV status**			
Living with HIV	69 (48.9)	20 (35.7)	0.063
Not living with HIV	64 (45.4)	34 (60.7)	
Unknown HIV status	0 (0.0)	1 (1.8)	
Prefer not to answer	8 (5.7)	1 (1.8)	
**HIV PrEP** ^¶¶^			
Yes	38 (45.8)	30 (76.9)	0.005
No	43 (51.8)	9 (23.1)	
Unknown	2 (2.4)	0 (0.0)	
HIV ARV***			
Not on ARV	6 (8.7)	1 (5.0)	0.724
Yes, nonadherent (missed ≥2 doses in previous 30 days)	28 (40.6)	7 (35.0)	
Yes, adherent	35 (50.7)	12 (60.0)	
**HIV status and PrEP/ARV use**			
HIV negative, not on PrEP	29 (21.8)	5 (9.3)	0.007
HIV negative, on PrEP	35 (26.3)	29 (53.7)	
HIV positive, not on ARV	6 (4.5)	1 (1.9)	
HIV positive, on ARV but nonadherent	28 (21.1)	7 (13.0)	
HIV positive, on ARV and adherent	35 (26.3)	12 (22.2)	
**Immunocompromising condition or medication** ^†††^			
Yes	14 (9.3)	3 (5.3)	0.441
No	130 (86.7)	53 (93.0)	
Don’t know/Prefer not to answer	6 (4.0)	1 (1.8)	
**STI history** ^§§§^	46 (30.7)	11 (19.3)	0.102
**Mpox vaccination status** ^¶¶¶^			
Not vaccinated	97 (72.9)	12 (23.5)	<0.001
Partially vaccinated	25 (18.8)	28 (54.9)	
Fully vaccinated	11 (8.3)	11 (21.6)	
**Close contact with someone who received an mpox diagnosis**			
Yes	109 (72.7)	41 (71.9)	0.827
No	22 (14.7)	10 (17.5)	
Unknown	19 (12.7)	6 (10.5)	
Close contact with someone who had symptoms consistent with mpox**** but no mpox diagnosis			
Yes	67 (44.7)	26 (45.6)	0.992
No	67 (44.7)	25 (43.9)	
Unknown	16 (10.7)	6 (10.5)	
**Intimate contact**			
Condomless receptive anal sex	58 (38.7)	11 (19.3)	0.008
Condomless insertive anal sex	55 (36.7)	10 (17.5)	0.008
Anal sex with a condom	12 (8.0)	3 (5.3)	0.497
Received oral sex without a condom	66 (44.0)	13 (22.8)	0.005
Gave oral sex without a condom	63 (42.0)	16 (28.1)	0.065
Gave or received oral sex with a condom	10 (6.7)	6 (10.5)	0.353
Close contact at a mass gathering where persons were partially undressed and touching	14 (9.3)	13 (22.8)	0.010
**Nonintimate contact**			
Provided in-home care	10 (6.7)	4 (7.0)	0.928
Shared food, utensils, or dishes	31 (20.7)	13 (22.8)	0.737
Shared towels, bedding, or clothing	49 (32.7)	10 (17.5)	0.031
Shared drug equipment	10 (6.7)	8 (14.0)	0.093

**Abbreviations**: ARV = antiretroviral; PrEP = preexposure prophylaxis; STI = sexually transmitted infection.

* Case- and control patients were recruited from the following 12 U.S. jurisdictions: California (excluding Los Angeles County), Colorado, Connecticut, Georgia, District of Columbia, Los Angeles County, Maryland, Minnesota, New York (excluding New York City), New York City, Oregon, and Tennessee.

^†^ Case-patients were identified or verified by jurisdiction health departments and had a confirmed or probable mpox or orthopoxvirus diagnosis on or after August 19, 2022.

^§^ Numbers might not sum to case- or control patient totals because of missing data. Percentages were calculated using nonmissing data.

^¶^ Control patients visited an STI, HIV care, or HIV PrEP clinic on or after August 19, 2022.

** P-values comparing the percentage of case-patients to control patients by sociodemographic and health categories were calculated using Pearson’s chi-square test. P-values for continuous variables were calculated using the Kruskal-Wallis test.

^††^ Participants reporting Hispanic ethnicity were categorized as Hispanic or Latino and might be of any race. The Other race category includes Asian, Native Hawaiian or other Pacific Islander, and American Indian or Alaska Native persons.

^§§^ Participants were asked to report the number of sexual partners they had had during the 3 weeks before completing the survey. The reported number was truncated at five to account for outliers and implausible values.

^¶¶^ HIV PrEP use was defined as use at time of survey and was calculated among persons who did not report living with HIV.

*** HIV ARV use was defined as use at time of survey and was calculated among persons who reported living with HIV; nonadherence was defined as missing ≥2 doses during the previous 30 days.

^†††^ Immunocompromising conditions were based on self-report and defined as having a medical condition that weakens the immune response, not including HIV, or taking a medicine that weakens the immune response.

^§§§^ Participants were asked to report STI diagnoses during the 3 weeks before completing the survey.

^¶¶¶^ Participants were categorized as not vaccinated if no reported doses were received on or before the index date. Participants were categorized as partially vaccinated if they received 1 dose ≥14 days before the index date and fully vaccinated if they received 2 doses ≥24 days apart (to allow for a 4-day window), with the second dose received ≥14 days before the index date. Participants who received their first vaccine dose ≤13 days before their index date were excluded.

**** Symptoms consistent with mpox included rash or skin lesions, fever, chills, headache, and muscle aches.

In the unadjusted models, behaviors associated with increased odds of mpox among persons reporting close contact with a person with mpox included condomless receptive anal sex (OR = 3.2; p = 0.006), condomless insertive anal sex (OR = 2.8; p = 0.009), receiving oral sex without a condom (OR = 2.7; p = 0.006), giving oral sex without a condom (OR = 2.0; p = 0.046), and sharing towels, bedding, or clothing (OR = 2.3; p = 0.034) (Table 2). After adjusting for age, race, HIV status and HIV PrEP or ARV use, number of sexual partners, mpox vaccination status, and index month, the only measured type of contact associated with mpox was condomless receptive anal sex. Adjusted odds of mpox were 5.4 times as high among those who reported having condomless receptive anal sex with a person with mpox than among participants who reported close contact with a person with mpox and no condomless receptive anal sex with that person (OR = 5.4; p = 0.031).

**TABLE 2:** Odds ratios of mpox associated with reported intimate and nonintimate close contact with a person with mpox — 12 jurisdictions, United States,* August 2022–July 2023

Characteristic	Unadjusted OR (95% CI) n = 207	p*-*value^†^	Adjusted OR (95% CI) n = 159	p*-*value^†^
**Age, yrs**	1.1 (1.0–1.1)	0.033	1.1 (1.0–1.2)	0.157
**Race and ethnicity** ^§^				
White, non-Hispanic	Ref	—	Ref	—
Black or African American, non-Hispanic	1.7 (0.7–3.8)	0.210	0.7 (0.1–3.5)	0.640
Hispanic or Latino	2.4 (1.0–5.4)	0.043	3.4 (0.7–16.5)	0.135
Other, non-Hispanic	1.1 (0.3–3.3)	0.904	0.2 (0–1.6)	0.123
**Gender identity**				
Male	Ref	—	—	—
Transgender female	2.6 (0.3–22.0)	0.375	—	—
Transgender male	—	—	—	—
Another gender identity	0.3 (0.1–1.4)	0.114	—	—
**No. of sexual partners** ^¶^	0.8 (0.7–1.0)	0.014	0.9 (0.6–1.3)	0.507
HIV status and PrEP/ARV use**				
HIV negative, not on PrEP	Ref	—	Ref	—
HIV negative, on PrEP	0.2 (0.1–0.6)	0.004	0.3 (0.1–2.2)	0.247
HIV positive, not on ARV	1.0 (0.1–11.2)	0.968	0.3 (0–19.0)	0.586
HIV positive, on ARV but nonadherent	0.7 (0.2–2.6)	0.588	0.4 (0–4.5)	0.469
HIV positive, on ARV and adherent	0.5 (0.2–1.7)	0.279	1.0 (0.1–8.3)	0.985
**Immunocompromising condition or medication** ^††^	1.8 (0.5–6.6)	0.393	—	—
**STI history** ^§§^	1.9 (0.9–4.0)	0.105	—	—
**Mpox vaccination status** ^¶¶^				
Not vaccinated	Ref	—	Ref	—
Partially vaccinated	0.1 (0–0.2)	<0.001	0 (0–0.1)	<0.001
Fully vaccinated	0.1 (0–0.3)	<0.001	0.1 (0–0.4)	0.005
**Intimate contact**				
Condomless receptive anal sex	3.2 (1.4–7.3)	0.006	5.4 (1.2–24.6)	0.031
Condomless insertive anal sex	2.8 (1.3–6.1)	0.009	1.0 (0.2–5.6)	0.980
Anal sex with a condom	1.6 (0.4–5.8)	0.510	0.5 (0–10.7)	0.661
Received oral sex without a condom	2.7 (1.3–5.4)	0.006	0.8 (0.2–3.4)	0.803
Gave oral sex without a condom	2.0 (1.0–4.1)	0.046	2.6 (0.6–11.2)	0.215
Gave or received oral sex with a condom	0.5 (0.2–1.6)	0.277	0.3 (0–3.7)	0.376
Close contact at a mass gathering where persons were partially undressed and touching	0.3 (0.1–0.7)	0.008	0.3 (0.1–1.3)	0.113
**Nonintimate contact**				
Provided in-home care	0.9 (0.3–3.2)	0.926	4.3 (0.2–79.9)	0.329
Shared food, utensils, or dishes	0.9 (0.4–2.0)	0.851	1.3 (0.3–5.5)	0.682
Shared towels, bedding, or clothing	2.3 (1.1–5.0)	0.034	1.7 (0.4–6.9)	0.480
Shared drug equipment	0.4 (0.2–1.2)	0.108	0.1 (0–1.1)	0.058

**Abbreviations**: ARV = antiretroviral; OR = odds ratio; PrEP = preexposure prophylaxis; Ref = referent group; STI = sexually transmitted infection.

* Case- and control patients were recruited from the following 12 U.S. jurisdictions: California (excluding Los Angeles County), Colorado, Connecticut, Georgia, District of Columbia, Los Angeles County, Maryland, Minnesota, New York (excluding New York City), New York City, Oregon, and Tennessee.

^†^ P-value calculated using logistic regression with a random intercept for jurisdiction.

^§^ Participants reporting Hispanic ethnicity were categorized as Hispanic or Latino and might be of any race. The Other race category includes Asian, Native Hawaiian or other Pacific Islander, and American Indian or Alaska Native persons.

^¶^ Participants were asked to report the number of sexual partners they had had during the 3 weeks before completing the survey.

** HIV PrEP use was defined as use at time of survey and was calculated among persons who did not report living with HIV. HIV ARV use was defined as use at time of survey and was calculated among persons who reported living with HIV; nonadherence was defined as missing ≥2 doses during the previous 30 days.

^††^ Immunocompromising conditions were based on self-report and defined as having a medical condition that weakens the immune response, not including HIV, or taking a medicine that weakens the immune response.

^§§^ Participants were asked to report STI diagnoses during the 3 weeks before completing the survey.

^¶¶^ Participants were categorized as not vaccinated if no reported doses were received on or before the index date. Participants were categorized as partially vaccinated if they received 1 dose ≥14 days before the index date and fully vaccinated if they received 2 doses ≥24 days apart (to allow for a 4-day window), with the second dose received ≥14 days before the index date. Participants who received their first vaccine dose ≤13 days before their index date were excluded.

## Discussion

Numerous studies have identified sexual contact as the primary risk factor for mpox; however, few have examined risk associated with specific intimate and nonintimate close contact behaviors. In this study, data from a previously conducted case-control study were analyzed to estimate the odds of mpox associated with selected intimate and nonintimate behaviors among MSM and transgender persons reporting close contact with a person with mpox. Condomless sex, including anal sex and oral sex, was associated with increased odds of mpox, as was sharing towels, bedding, and clothing. After adjusting for measured confounders, including mpox vaccination and concurrent close contact behaviors, condomless receptive anal sex with a person with mpox remained associated with increased odds of mpox. Although condoms might reduce MPXV exposure at anogenital or oral mucosal sites, condoms alone might not prevent all exposures to MPXV because rash can occur on other parts of the body and transmission can occur through other routes, including saliva and respiratory secretions (*5*).

As clade II mpox continues to circulate in the United States, mpox mitigation activities remain critical (*6*). The mpox vaccine is highly effective (*4*,*7*,*8*) and remains an important tool in interrupting the spread of mpox. However, only one in four of the approximately two million persons eligible to receive the vaccine in the United States has received both doses. A multifaceted approach to reducing mpox transmission risk remains crucial to preventing large outbreaks. In addition to vaccination, clinicians should educate patients about using safer sex strategies to reduce exposure to MPXV, talking with sex partners about any mpox signs or symptoms, being aware of any unexplained rashes or lesions on a partner’s body, and avoiding close or intimate contact if they or a sex partner become infected with MPXV or experience an mpox-like rash.^§§§^

Studies from areas with endemic mpox and during the ongoing 2022 global outbreak have identified contaminated household items such as linens and utensils as potential, albeit less common, MPXV transmission routes (*3*,*9*,*10*). In this study, sharing bedding, towels, or clothing with a person with mpox was associated with acquiring mpox in the unadjusted but not the adjusted analysis. In addition to contaminated household items, shared bedding during sex might contribute to transmission; more studies are needed to better understand transmission pathways.

### Limitations

The findings in this report are subject to at least five limitations. First, selection bias is likely because survey participation was voluntary and recruitment for control patients occurred in sexual health, HIV care, or HIV PrEP clinics. Differences in sexual risk-taking behaviors might exist between those who participated in the survey and those who did not and between persons who did and did not seek health care. Second, survey data were self-reported and might be subject to social desirability or recall bias, particularly because of the sensitive nature of some of the questions regarding sexual behaviors, and because the time between index event and survey completion varied. Third, intimate contact and sexual behavior variables were limited to a few broad measures in this study and do not account for potentially important factors such as frequency and duration of contact, partner type, group sex, substance use, or impact of sexual networks, all of which might affect risk for mpox transmission. Fourth, <15% of survey participants reported close contact with a person with mpox; because of this small sample size, the analysis might be underpowered to detect associations with behaviors that were less commonly reported. Finally, although the 12 U.S. jurisdictions included in this study covered a broad geographic area, data might not be generalizable to the entire U.S. population.

### Implications for Public Health Practice

The mpox vaccine is highly effective, and clinicians should continue to promote vaccination among eligible persons.^¶¶¶^ In addition, results from this study indicate that implementation of multiple prevention approaches, including education on safer sex practices, might further reduce risk.

## Footnotes

* Case- and control patients were recruited from the following 12 U.S. jurisdictions: California (excluding Los Angeles County), Colorado, Connecticut, District of Columbia, Georgia, Los Angeles County, Maryland, Minnesota, New York (excluding New York City), New York City, Oregon, and Tennessee.

† Sexually active was defined as having one or more sexual partner since August 1, 2022.

§ The definition of MSM used in the parent study and this analysis included 1) participants who reported being male and described their sexual identity as gay, bisexual, a different term, or preferred not to answer, or 2) participants who reported being male, described themselves as straight, and responded that they had sex with men.

¶ Mpox exposure history was assessed by asking participants if, during the 3 weeks before their index date, they had exposure to 1) a person with diagnosed mpox, or 2) a person with symptoms consistent with mpox (e.g., rash or skin lesions, fever, chills, headache, or muscle aches) but who did not receive a diagnosis of mpox. The exposure might have occurred after the contact received a diagnosis or developed mpox symptoms or during the 3 weeks before diagnosis or symptom onset.

** Types of intimate contact included condomless receptive anal sex; condomless insertive anal sex; anal sex with a condom; vaginal sex, with or without a condom; receiving oral sex without a condom; giving oral sex without a condom; giving or receiving oral sex with a condom; other intimate contact (e.g., cuddling, kissing, touching partner's genitals or anus, or sharing sex toys); and close contact at a mass gathering where persons were partially undressed and touching (e.g., raves, pool parties, or dance events).

†† Types of nonintimate contact included providing in-home care to a person with diagnosed mpox; sharing food, utensils, or dishes; sharing towels, bedding, or clothing; sharing drug equipment (e.g., needles, cookers, or bongs); and face-to-face contact (being within 6 ft of an unmasked person with mpox for >3 hours while not wearing a mask).

§§ Immunocompromising conditions were based on self-report and defined as having a medical condition that weakens the immune response (other than HIV) or taking a medication that weakens the immune response.

¶¶ Participants were asked to report the number of sexual partners they had had during the 3 weeks before completing the survey. The reported number was truncated at five to account for outliers and implausible values.

*** Participants were categorized as not vaccinated if no reported doses were received on or before the index date. Participants were categorized as partially vaccinated if they received 1 dose ≥14 days before the index date and fully vaccinated if they received 2 doses ≥24 days apart (to allow for a 4-day window), with the second dose received ≥14 days before the index date. Participants who received their first vaccine dose ≤13 days before their index date were excluded.

††† 45 C.F.R. part 46.102(l)(2), 21 C.F.R. part 56; 42 U.S.C. Sect. 241(d); 5 U.S.C. Sect. 552a; 44 U.S.C. Sect. 3501 et seq.

§§§ https://www.cdc.gov/mpox/hcp/clinical-signs/index.html

¶¶¶
https://www.cdc.gov/poxvirus/mpox/vaccines/index.html
